# Dataset on student experiences and perceptions of Emergency Remote Teaching (ERT) in an Irish University

**DOI:** 10.1016/j.dib.2022.107954

**Published:** 2022-02-14

**Authors:** Gearóid Ó. Súilleabháin, Tom Farrelly, Sean Lacey

**Affiliations:** a Department of Technology Enhanced Learning, Munster Technological University, Bishopstown, Cork, Co Cork, Ireland; b Department of Social Sciences, Munster Technological University, Tralee, Co Kerry, Ireland; c Office of Research Integrity & Compliance, Munster Technological University, Bishopstown, Cork, Co Cork, Ireland

**Keywords:** Online learning, Emergency Remote Teaching, Remote teaching, COVID-19, Student experience

## Abstract

This paper presents a dataset from a survey of student experiences and perceptions of Emergency Remote Teaching (ERT) during the COVID-19 pandemic in the Munster Technological University, a new higher education institute in the south-west of Ireland formed from the recent merger of two older institutes of technology. Data were collected throughout the month of May 2021 using the online survey tool Typeform with the survey itself being promoted with the student body primarily via email, social media and the university's Learning Management System. There were a total of 1703 responses (approx. 11% response rate which maps to less than a 2.5% margin of error at a 95% level of confidence). The survey was designed to elicit data with respect to student background, workspace and access; ERT tools and platforms used; challenges faced during the ERT period as well as benefits and preferred future teaching and learning modes. The dataset could be of interest to other researchers, policy makers and administrators interested in student experiences of and insight with respect to ERT and student views on the legacy of ERT. Data also provide opportunity to cross-tabulate key variables such as, e.g., internet connectivity and overall ERT experience. Survey questions are also included for replicability purposes. The data article also includes students’ responses received in CSV and formatted spreadsheet formats.

## Specifications Table

**Table:** 

Subject	Education
Specific subject area	Student Experiences and Perceptions of Emergency Remote Teaching
Type of data	Figures Dataset in CSV and MS Excel format Survey Questions in MS Word Format
How data were acquired	Data acquired by distributing a link to an online survey to university students via email, Learning Management System and Student Union social media channels.
Data format	Filtered Raw
Parameters for data collection	The data were gathered via an online survey designed to elicit student responses with respect to experiences and perceptions of Emergency Remote Teaching (ERT) during the COVID-19 pandemic. Survey respondents are students – undergraduate and postgraduate – based across a number of campuses of the newly-formed Munster Technological University in the south west of Ireland who have been studying online and at a distance since the effective closure of physical campuses in March 2020.
Description of data collection	Data were collected throughout the month of May 2021 using the online survey tool Typeform with the survey itself being promoted with the student body primarily via email, social media and the university's Learning Management System.
Data source location	Institution: Munster Technological University City/Town/Region: Munster Country: Ireland
Data accessibility	Figures with the article Dataset as follows: Repository name: South West Open Research Deposit (SWORD) Data identification number: https://doi.org/10.34719/wsrs-0j15 Direct URL to data: https://sword.cit.ie/open_research/1/

## Value of the Data

• The data presented in this article is useful for researchers interested in understanding the experiences of students who transitioned to Emergency Remote Teaching (ERT) as part of their university's response to the cessation of on-campus teaching and learning activities during the COVID-19 emergency.
• The data should be of benefit to policy makers, researchers, administrators and heads of function to gain further insights and make decisions around how best to reap the benefits and avoid the potential pitfalls of ERT and related practices in the future.
• The raw data responses could be used to investigate and test hypotheses of the relationships between student experiences and/or perceptions in relation to ERT.
• The data could be used in comparative studies that make use of similar data sets from other higher education institutes and other education and training organisations.
• Data is also important in view of the possibility of other future possible disruptions to on-campus teaching and learning activity, be these local, regional, national or global.
• The data may, in addition, guide practitioners of online and/or blended learning in their choice of approach to technology for teaching, learning and assessment, in addition to support models for students. Furthermore, the data may help student services better understand the challenges students face and their preferences with respect to online and/or blended learning implementations.
• The data presented could more broadly be used to inform the formulation of policies and the identification of strategic priorities with respect to the integration of online and/or digital learning as an additional component to complement the face-to-face campus experience.

## 1 Data Description

The widespread closure of higher education campuses in response to the COVID-19 pandemic obliged higher education institutes the world over to transition quickly to what has since commonly become known as Emergency Remote Teaching (ERT), i.e., a “a temporary shift of instructional delivery to an alternate delivery mode due to crisis circumstances” [1]. This transition was unprecedented in its scale and has had profound effects, not least on the lives and learning experiences of higher education students [2]. Concomitantly, extensive research has been devoted to investigating the phenomenon of ERT with a particular sub-category of the emergent literature being devoted to the perspectives, insights, experiences and issues of higher education students over this period [3].

The data shared in this article were obtained from 1703 students of the Munster Technological University, all of whom were studying remotely as part of the university's transition to ERT. Data were gathered via an online survey designed to illicit student responses with respect to their experiences of ERT over this time.

The data provided consists of: the survey questions in MS Word format, the responses obtained in CSV and MS Excel spreadsheet formats and some charts and data visualisations (in article).

**Fig. 1:**
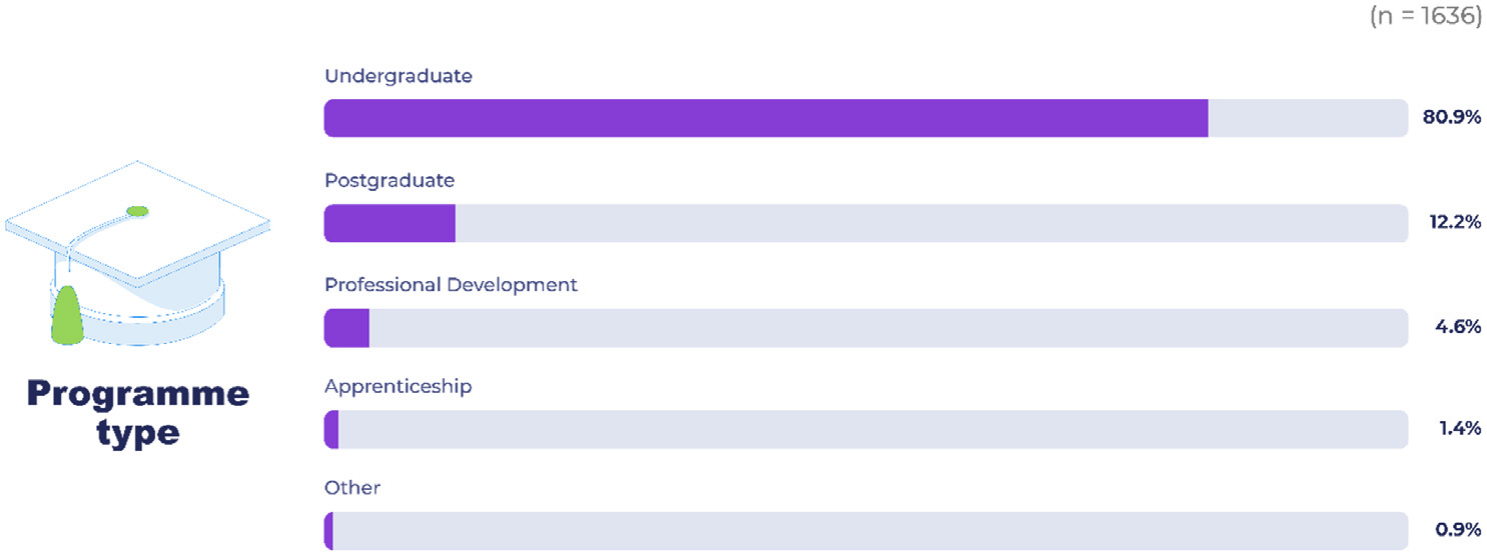
Type of programme.

As shown in Fig. 1, respondents were predominantly undergraduate students, but the sample also included postgraduate, professional development, apprenticeship and “other” students, in a way broadly representative of the overall university student distribution.

**Fig. 2:**
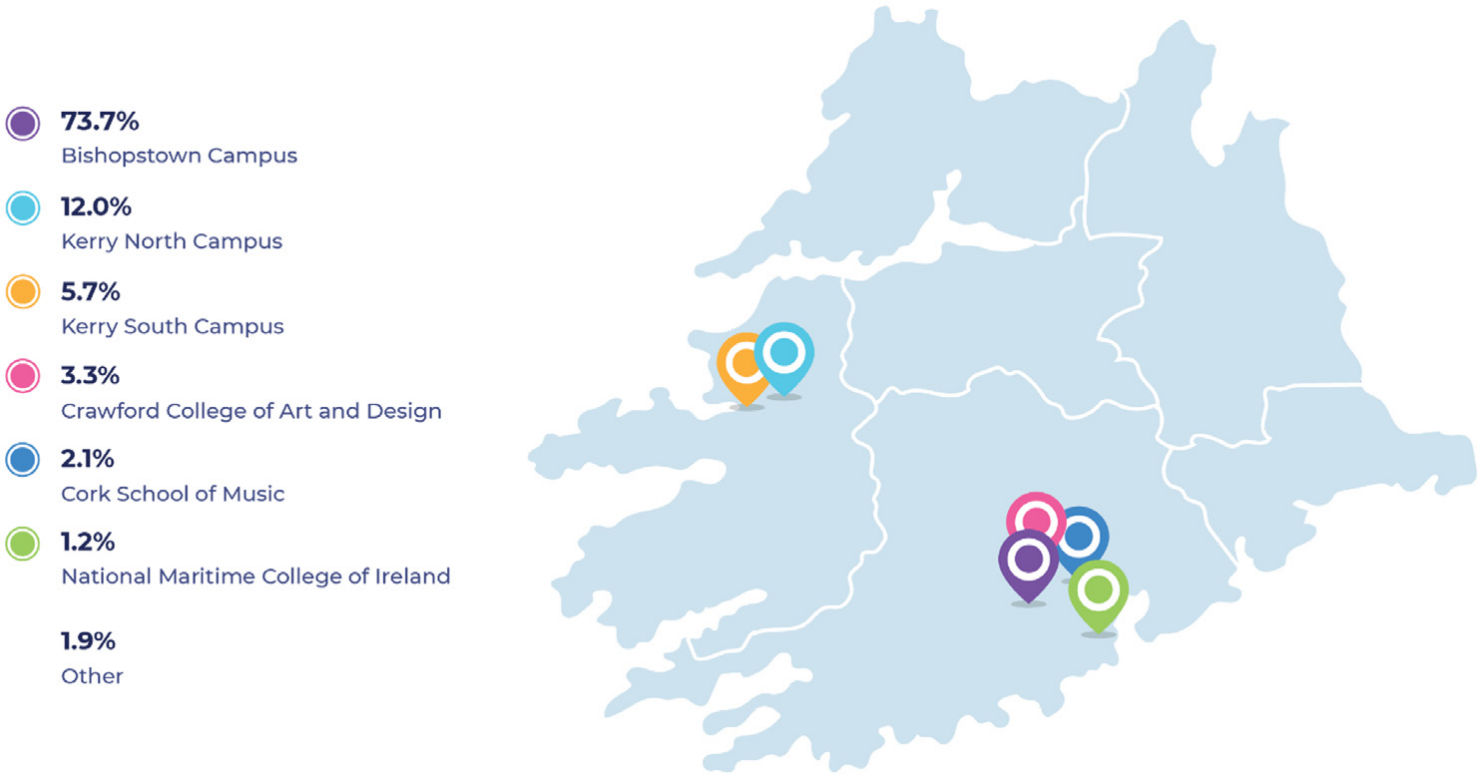
University campuses where respondents would usually attend.

As illustrated in Fig. 2, the majority of respondents are either based on the Bishopstown Campus (73.7%) in Cork city, Co Cork or the Kerry North Campus, Tralee, Co Kerry (12.0%). Sample distribution closely matches the overall university student distribution.

**Fig. 3:**
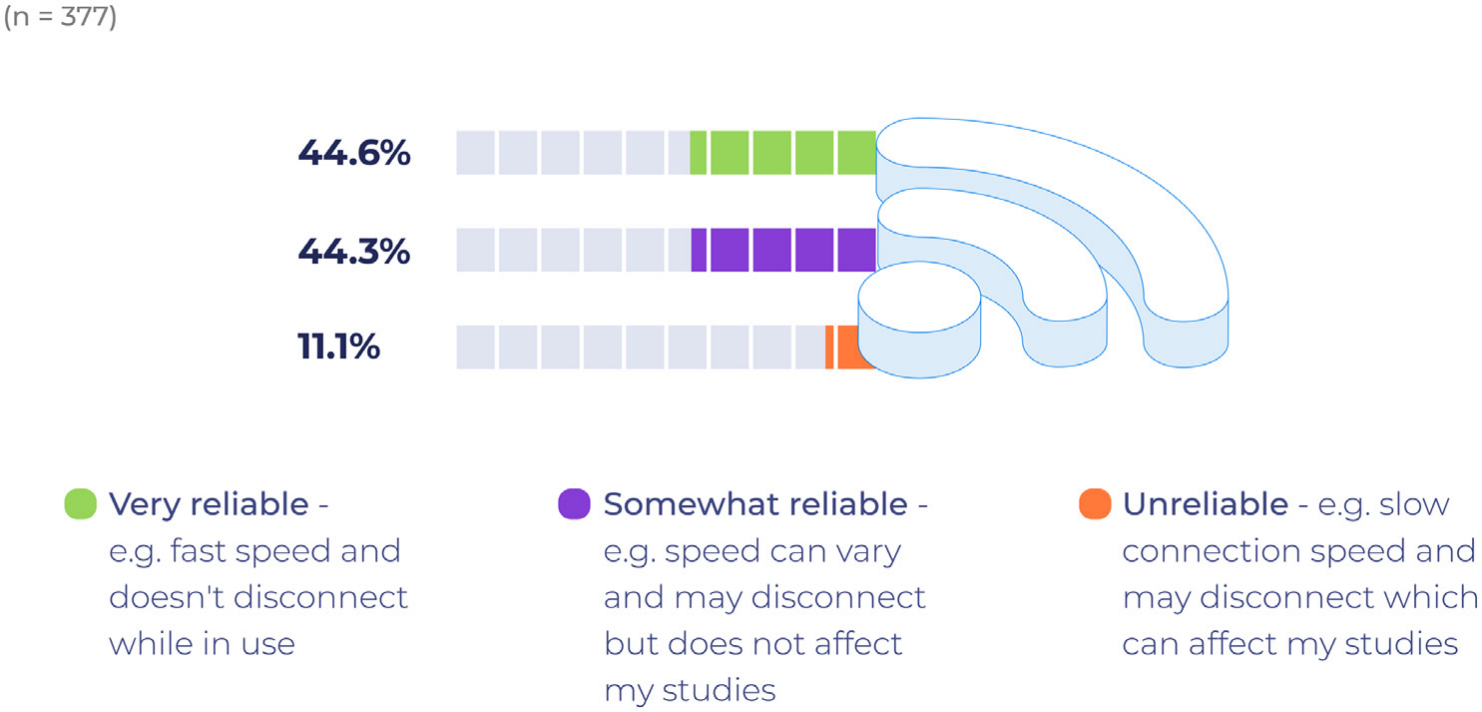
Internet connectivity.

As Fig. 3 illustrates, only one third of the respondents characterise their internet connection as being ‘very reliable’; with half reporting that their connection is ‘somewhat reliable’ with the remainder reporting unreliable connection speeds and loss of connection to such an extent that it affects their studies.

**Fig. 4:**
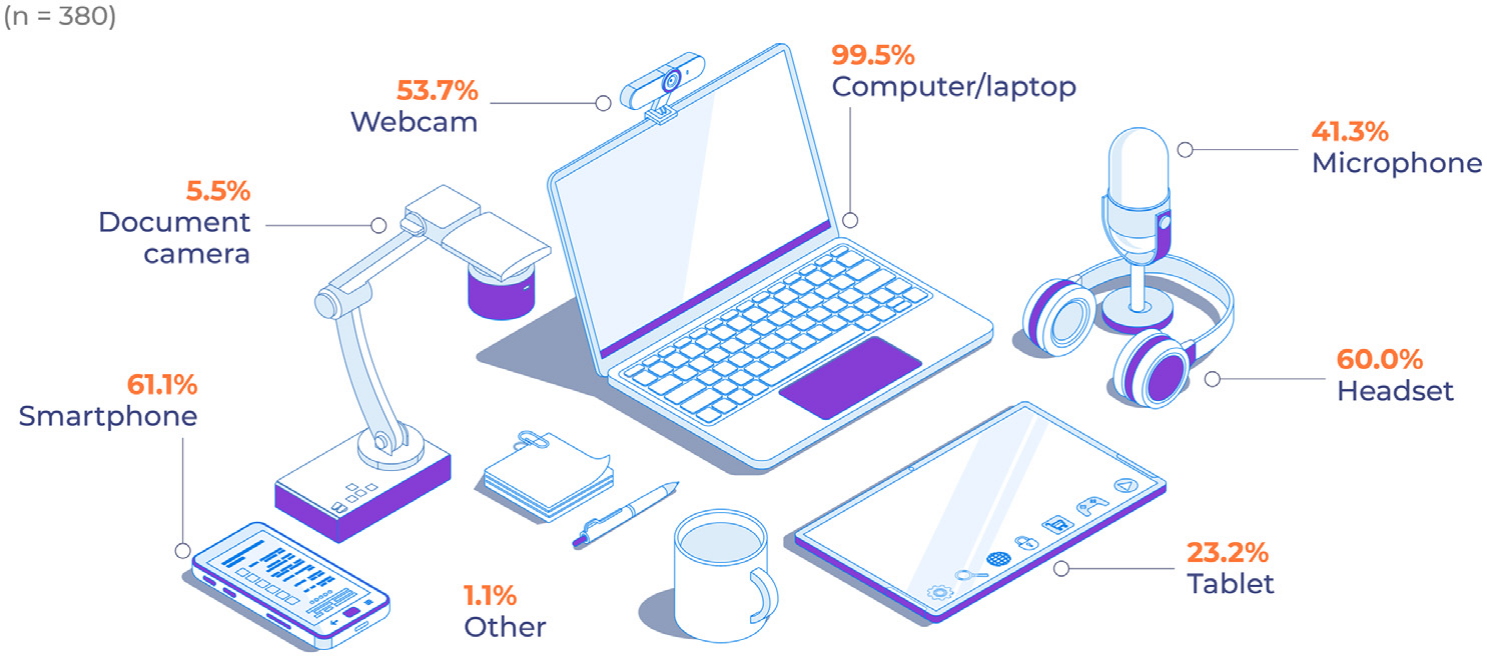
Access to suitable hardware and peripherals.

As can be seen from Fig. 4, 98.2% of respondents have access to a computer or laptop while 77.6% have access to a smartphone. Just over half of respondents (56.9% and 53.2%, respectively) indicate they have access to a stand-alone webcam or microphone. Less than half of respondents have access to other peripherals such as a headset (42.5%) or tablet (21.8%).

## 2 Experimental Design, Materials and Methods

The dataset was gathered using an online survey. The shared data cover 12 of the survey questions which broadly relate to: students background and demographic data; student ERT experiences and engagement and, finally, preferences with respect to the continuation of possible ERT benefits and future teaching and learning modes. Logic branching was used for one of the survey questions (“Do you feel there were any benefits associated with remote learning?”) which skipped two subsequent questions for respondents answering in the negative. Two final questions of the original survey gathered qualitative data – these qualitative data are not shared as part of this article submission.

The survey was distributed via the ‘allstudents’ email list and via the university Learning Management System (LMS). A reminder email was sent out one week after the initial email . The survey link remaining open for fourteen days in total. The survey was also distributed through Student Union social media accounts (Student Union Facebook and Twitter). The latter distribution channel opens up the possibility that outside parties who are not registered students of the university could in theory have responded to the survey. Responses to a question looking for confirmation of the campuses respondents usually attend suggests this did not transpire however, as does the fact that the demographic data of the sample matches well with characteristics of the overall population. Qualitative responses – analysed separately to this article – also show no evidence of malicious or disruptive intent.

The online survey/form solution employed is called “Typeform” which offers basic tools to automatically collate, analyse and visualise survey responses. Summary data from this platform was used as the basis for the various visualisations contained in this article. The survey platform also allows for the export of a CSV version of all student responses which is the key data this article presents.

No further analysis has been conducted on the data at this time.

## Ethics Statement

As survey respondents could be directed from multiple sources a participant information letter was linked from the front page of the survey which all respondents had to view before beginning the survey. Provision of the information letter in this way meant all respondents were able to make an informed decision about their consent to proceed with the survey. Respondents, furthermore, were able to withdraw from the survey at any point before completing by simply closing the survey link in their browser or by not clicking on the proceed option. If respondents did proceed with the survey, they were deemed to have given consent upon clicking on the final “Submit” button (as indicated in the participant information letter). At the start of the survey, respondents were asked to confirm their age and those under 18 were informed as follows and not able to proceed with the survey: “This survey is intended for students who are 18 years or older. Unfortunately, your age does not permit you to participate.” Ethical approval was granted for this research by the Institute Research Ethics Committee (IREC) on the 13/05/2021.

## Declaration of Competing Interest

The authors declare that they have no known competing financial interests or personal relationships that could have appeared to influence the work reported in this paper.

## Data Availability

Dataset on Student Experiences and Perceptions of Emergency Remote Teaching (ERT) in an Irish University (Original data) (South West Open Research Deposit (SWORD)).
